# Supplementary material for: German-Wide Interlaboratory Study Compares Consistency, Accuracy and Reproducibility of Whole-Genome Short Read Sequencing
Source: Front Microbiol. 2020 Sep 11;11:573972. doi: 10.3389/fmicb.2020.573972 (PMC7516015; doi:10.3389/fmicb.2020.573972)
Supplement: FILE S1 — Information about the strains used for the interlaboratory study. [file Data_Sheet_1.zip › Supplementary File 1.DOCX]

**General information about the strains used for the interlaboratory study**

| **Sample** | **Organism** | **Serovar** | **MLST** |
| --- | --- | --- | --- |
| 19-RV1-P64-1 | *Campylobacter jejuni* |  | 4774 |
| 19-RV1-P64-2 | *Campylobacter jejuni* |  | 21 |
| 19-RV1-P64-3 | *Listeria monocytogenes* | IIc | 9 |
| 19-RV1-P64-4 | *Listeria monocytogenes* | IIb | 59 |
| 19-RV1-P64-5 | *Salmonella enterica* subsp*. enterica* | Infantis | 32 |
| 19-RV1-P64-6 | *Salmonella enterica* subsp. *enterica* | Paratyphi B var. Java | 28 |

**Information about the uncirculated PacBio sequences used as reference sequences for SNP calling**

| **Sample** | **Length** | **Number of contigs** | **GC** |
| --- | --- | --- | --- |
| 19-RV1-P64-1 | 1,619,699 bp | 1 | 30.53 % |
| 19-RV1-P64-2 | 1,716,034 bp | 1 | 30.51 % |
| 19-RV1-P64-3 | 3,000,034 bp | 2 | 37.96 % |
| 19-RV1-P64-4 | 3,019,943 bp | 1 | 37.93 % |
| 19-RV1-P64-5 | 5,052,870 bp | 2 | 51.98 % |
| 19-RV1-P64-6 | 4,789,411 bp | 4 | 52.22 % |

**Antimicrobial resistance genes and plasmid markers identified from the uncirculated PacBio sequences**

| **Sample** | **Number of plasmid markers** | **Plasmid markers** | **Number of resistance genes** | **Resistance genes** |
| --- | --- | --- | --- | --- |
| 19-RV1-P64-1 | 0 |  | 2 | blaOXA-184;tet(O) |
| 19-RV1-P64-2 | 0 |  | 1 | blaOXA-605 |
| 19-RV1-P64-3 | 0 |  | 2 | fosX;lin |
| 19-RV1-P64-4 | 0 |  | 2 | fosX;lin |
| 19-RV1-P64-5 | 3 | IncHI2_1;TrfA_1;IncHI2A_1 | 13 | sul1;qacEdelta1;aadA1;ere(A);sul1;qacEdelta1;aadA1;blaVIM-1;catA1;blaACC-1;sul1;aph(6)-Id;aph(3'')-Ib |
| 19-RV1-P64-6 | 1 | IncI1_1_Alpha | 9 | aadA1;aadA1;dfrA1;aadA1;dfrA1;sul2;blaTEM-1;dfrA1;tet(A) |
