# Supplementary material for: German-Wide Interlaboratory Study Compares Consistency, Accuracy and Reproducibility of Whole-Genome Short Read Sequencing
Source: Front Microbiol. 2020 Sep 11;11:573972. doi: 10.3389/fmicb.2020.573972 (PMC7516015; doi:10.3389/fmicb.2020.573972)
Supplement: FILE S1 — Information about the strains used for the interlaboratory study. [file Data_Sheet_1.zip › Supplementary File 2.DOCX]

**Ergebnisprotokoll für den 1. §64 LFGB Ringversuch**

**„NGS-Bakteriencharakterisierung (2019)“**

durchgeführt durch das Bundesinstitut für Risikobewertung
Studienzentrum für Genomsequenzierung und –analyse
Abteilung Biologische Sicherheit

Code-Nr. des Laboratoriums :

Name des Laboratoriums :

Empfangsdatum der Ringversuchsproben :

Beginn des Ringversuches :

1. **Transport**
   1. **Wann kam das Paket mit den Ringversuchsproben an?**

Datum:

- 1. **War das Paket beschädigt?**

Ja/Nein:

1. **DNA Konzentrations- und Qualitätsbestimmung**

- 1. **Welche Methode wurde für die DNA Konzentrationsbestimmung**

**angewandt?**

• Qubit
 • Nanodrop
 • keine
 • andere:

 Falls erfasst: Bitte tragen Sie die DNA Konzentration in die Excel-
 Ergebnisstabelle ein.

- 1. **Haben Sie die DNA Qualität anhand der Bestimmung von Absorptionsverhältnissen (A260/A280 und A260/A230) geprüft?**

• Ja, mittels Nanodrop
 • Ja, mittels:
 • Nein

Falls erfasst: Bitte tragen Sie die ermittelten Werte in die Excel-Ergebnisstabelle ein.

- 1. **Wurde die Qualität (z.B. Degradation) der DNA mit
     Gelelektrophorese/Bioanalyzer/Fragment Analyzer überprüft?**

Ja / Nein

Falls Ja: Bitte vermerken Sie in der Excel-Ergebnisstabelle
 für welche DNA Proben eine Degradation beobachtet wurde.

1. **Bibliothekenherstellung (Library Prep)**
   1. **Welche Kits haben Sie für die Vorbereitung der Library verwendet? Bitte geben Sie den Namen und die Bestellnummer der verwendeten Kits an.**

Name:
Bestellnummer:

- 1. **Wie viel DNA haben Sie zu Beginn eingesetzt?**

Bitte tragen Sie die DNA Mengen [ng] in die Excel-Ergebnisstabelle ein.

- 1. **Erforderte Ihr Protokoll die enzymatische oder mechanische Fragmentierung der DNA?**

• enzymatisch
 • mechanisch
 • andere:

- 1. **In welchem Umfang folgten Sie dem Kit Protokoll?**

• vollständig

• kleine Modifikationen

welche:

• größere Modifikationen
 welche:

- 1. **Welche Methode wurde zum Überprüfen der Qualität/Fragmentlänge der erzeugten Bibliothek verwendet?**

• Bioanalyzer/ Fragment Analyzer

• Gelelektrophorese

• keine

• andere:

- 1. **Haben Sie die DNA Bibliotheken normalisiert?**

Ja / Nein

Falls ja, über welche Methode:

• manuell über die Konzentration und Fragmentlänge
 mit Konzentrationsbestimmung über:
 • Qubit
 • qPCR
 • Bead-basiert
 • andere:

- 1. **Wie wurde die DNA gepooled?**

• alle Proben gleich
 • gewichtet gemäß der Genomgröße der unterschiedlichen
 Spezies
 • andere:

- 1. **Was war die Ausgangsgesamtgröße aller sequenzierten Genome in
     Millionen Basenpaaren (Mb) in ihrem Sequenzierungslauf*?**

Gesamtgröße [Mb]:

* Hinweis: 1 *Salmonella* Genom: ca. 5 Mb

1 *Campylobacter* Genom: ca. 1,7 Mb

1 *Listeria* Genom: ca. 3 Mb

2 x *Salmonella*, 2 x *Listeria*, 2 x *Campylobacter*: 19,4 Mb

24 x Salmonella: 120 Mb

etc…

- 1. **Welche Library-Menge haben Sie für die Sequenzierung eingesetzt?**

Illumina- manuelle Normalisierung:
 • Library-Ladekonzentration [pM]:
 Illumina- bead-basierte Normalisierung
 • Volumen des Library Pools [µl]:
 Ion Torrent (Ladekonzentration für emPCR)
 • Library-Ladekonzentration [pM]:
 • weitere relevante Angaben:

1. **Sequenzierung**
   1. **Welche Sequenzierungsplattform nutzen Sie?**

Illumina
• iSeq
• MiniSeq
• MiSeq series
• NextSeq series
• HiSeq series
• NovaSeq series
Ion Torrent
• PGM
• Proton
• S5/S5XL
andere:

- 1. **Verwenden Sie eine Sequenzierungskontrolle?**(z.B. PhiX, Test Fragments, Standard-DNA, definierte Isolat-DNA, etc.)

Ja/Nein

Falls ja, welche:

- 1. **Welches Kit verwenden Sie für die Sequenzierung der Proben? Bitte geben Sie den Namen und die Bestellnummer des verwendeten Kits an.**

Name:
Bestellnummer:

- 1. **Welche Sequenzierungstiefe (x-fache Abdeckung der Genome) streben Sie in der Regel an?**

Sequenzierungstiefe:

- 1. **Weitere Fragen für die Sequenzierung mittels Illumina Geräten**
     1. **Sequenzieren Sie single-end oder paired-end?**

• single-end
• paired-end

- - 1. **Wie viele Zyklen sequenzieren Sie?**(Für die paired-end Sequenzierung bitte mit 2x angeben, z.B. 2x151 Zyklen)

Zyklenzahl:

- - 1. **Welche Cluster-Dichte haben Sie erreicht?**

K/mm2:

- - 1. **Wie groß war die Prozentzahl an Basen mit einem Qualitäts-Score >=30?**

>=Q30%:

- - 1. **Wie hoch war die Gesamtausbeute an sequenzierten Basen (total yield [G])?**

Total yield [G]:

- - 1. **Nennung weiterer relevanter Qualitätsparameter, die Sie zur Qualitätsprüfung eines Laufes heranziehen:**

- 1. **Weitere Fragen für die Sequenzierung mittels Ion Torrent Geräten**
     1. **Wie hoch war ihre ISP Density und wie viele ISPs waren polyklonal?**

ISP Density:
polyklonal [%]:

- - 1. **Wie groß ist die totale Readanzahl (total reads), der Prozentsatz an verwendbaren Reads und die durchschnittliche Readlänge?**

Total reads:
 usable reads (%):
 durchschnittliche Readlänge:

- - 1. **Nennung weiterer relevanter Qualitätsparameter, die Sie zur Qualitätsprüfung eines Laufes heranziehen:**

1. **Anmerkungen zur Ringversuchsdurchführung**

Anmerkungen zu Faktoren und Problemen, die das Ergebnis beeinflusst haben könnten:

1. **Anmerkungen zur Ringversuchsorganisation und Verbesserungsvorschläge**

• Probenversand:
• Ergebnisdokumentation:
• Datenübermittlung:
• Sonstiges:
