# Supplementary material for: German-Wide Interlaboratory Study Compares Consistency, Accuracy and Reproducibility of Whole-Genome Short Read Sequencing
Source: Front Microbiol. 2020 Sep 11;11:573972. doi: 10.3389/fmicb.2020.573972 (PMC7516015; doi:10.3389/fmicb.2020.573972)
Supplement: FILE S1 — Information about the strains used for the interlaboratory study. [file Data_Sheet_1.zip › Supplementary File 7.PDF]

LC02a

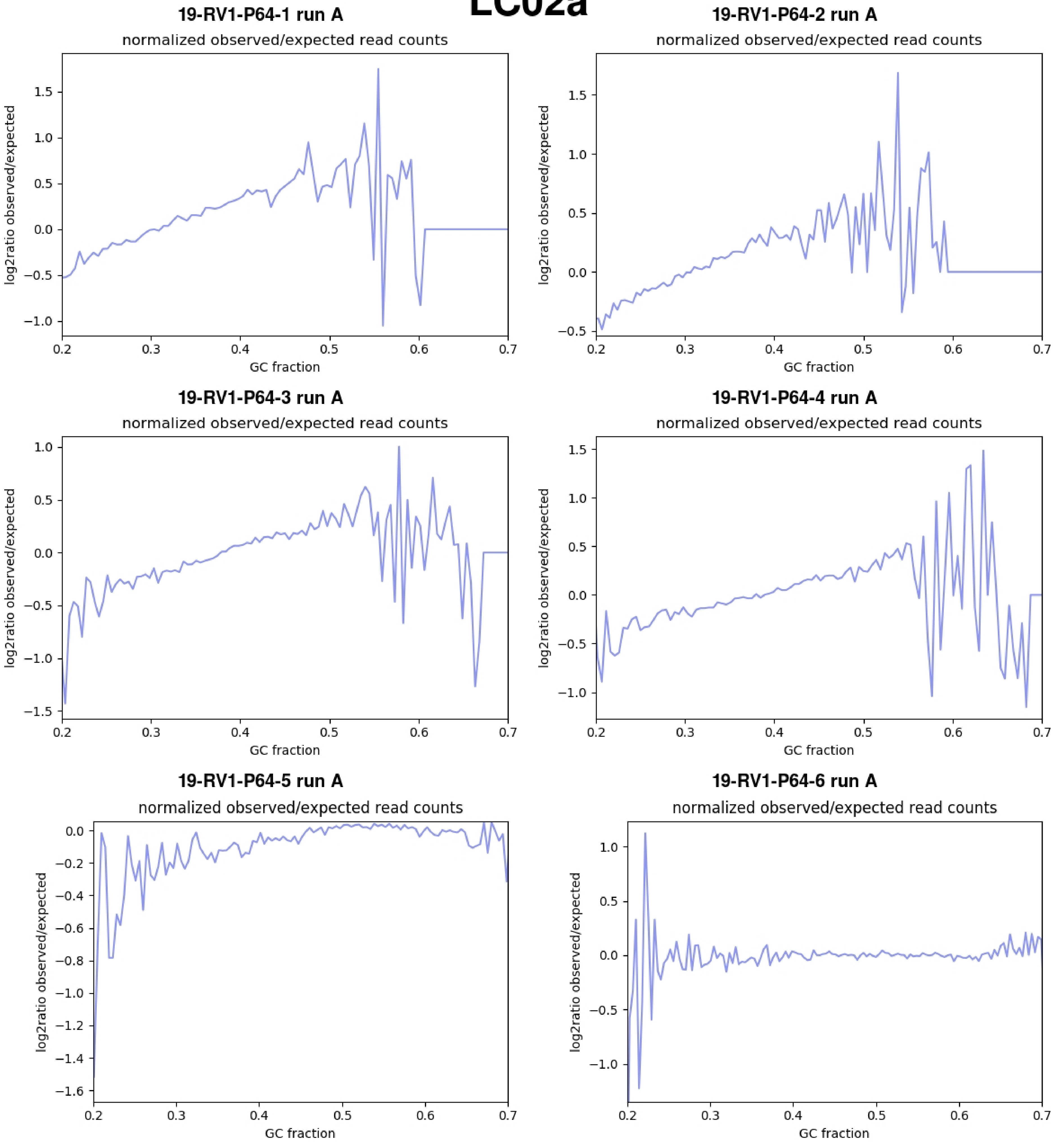
19-RV1-P64-1 run A
normalized observed/expected read counts
19-RV1-P64-2 run A
normalized observed/expected read counts
19-RV1-P64-3 run A
normalized observed/expected read counts
19-RV1-P64-4 run A
normalized observed/expected read counts
19-RV1-P64-5 run A
normalized observed/expected read counts
19-RV1-P64-6 run A
normalized observed/expected read counts
log2ratio observed/expected
GC fraction

LC02b

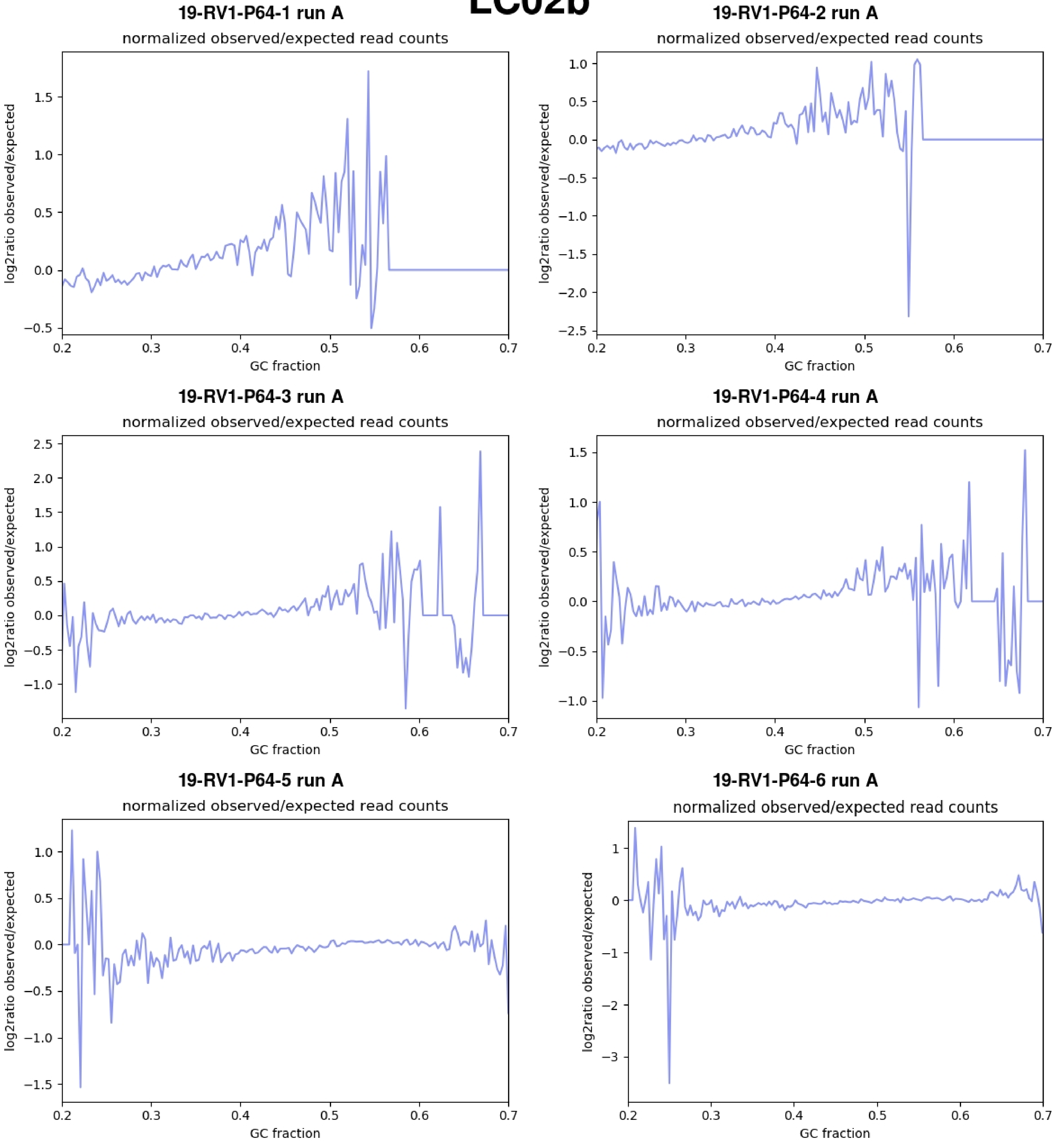

19-RV1-P64-1 run A
normalized observed/expected read counts
log2ratio observed/expected
GC fraction
19-RV1-P64-2 run A
normalized observed/expected read counts
log2ratio observed/expected
GC fraction
19-RV1-P64-3 run A
normalized observed/expected read counts
log2ratio observed/expected
GC fraction
19-RV1-P64-4 run A
normalized observed/expected read counts
log2ratio observed/expected
GC fraction
19-RV1-P64-5 run A
normalized observed/expected read counts
log2ratio observed/expected
GC fraction
19-RV1-P64-6 run A
normalized observed/expected read counts
log2ratio observed/expected
GC fraction

LC02c

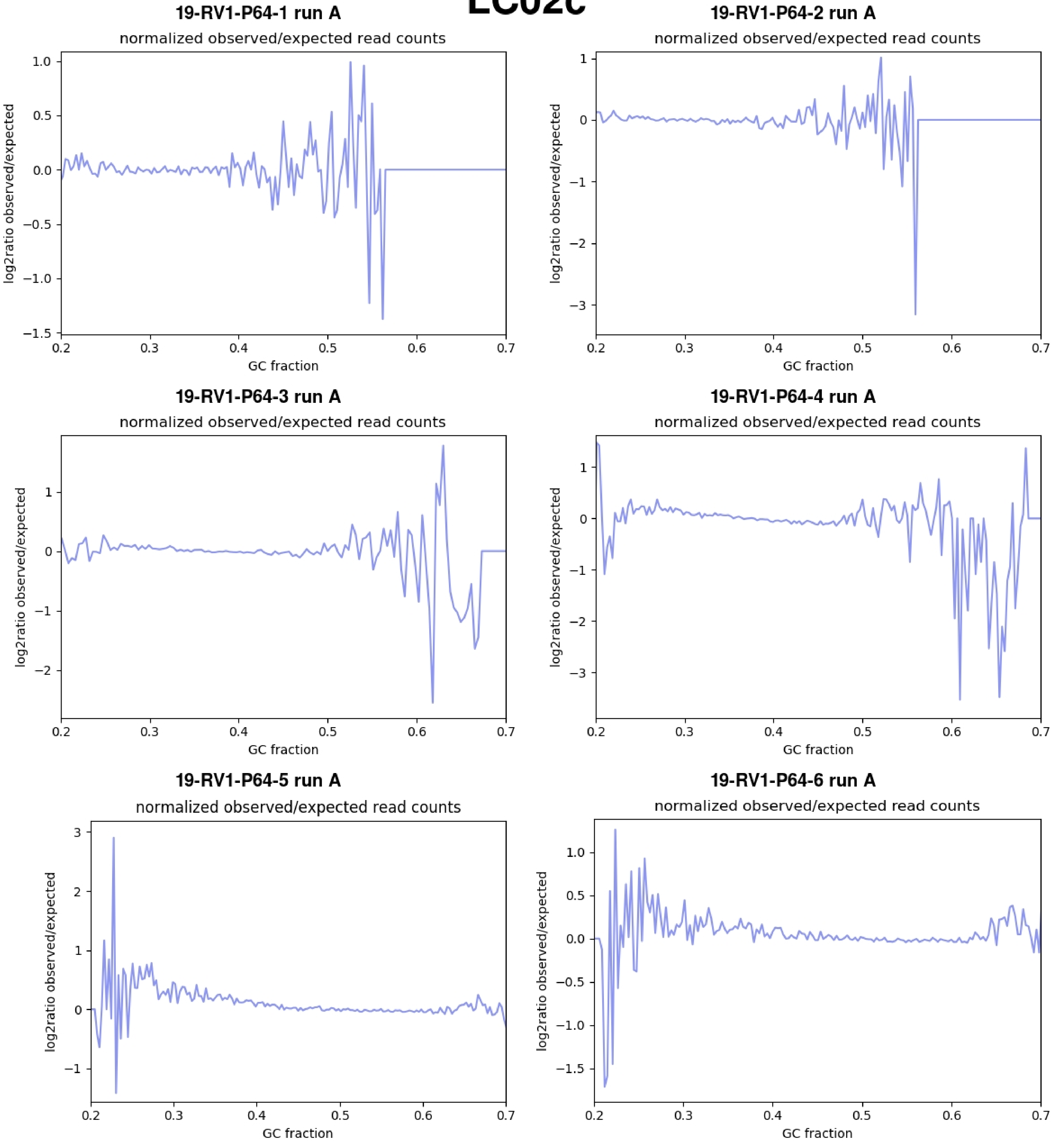
19-RV1-P64-1 run A
normalized observed/expected read counts
log2ratio observed/expected
GC fraction
19-RV1-P64-2 run A
normalized observed/expected read counts
log2ratio observed/expected
GC fraction
19-RV1-P64-3 run A
normalized observed/expected read counts
log2ratio observed/expected
GC fraction
19-RV1-P64-4 run A
normalized observed/expected read counts
log2ratio observed/expected
GC fraction
19-RV1-P64-5 run A
normalized observed/expected read counts
log2ratio observed/expected
GC fraction
19-RV1-P64-6 run A
normalized observed/expected read counts
log2ratio observed/expected
GC fraction

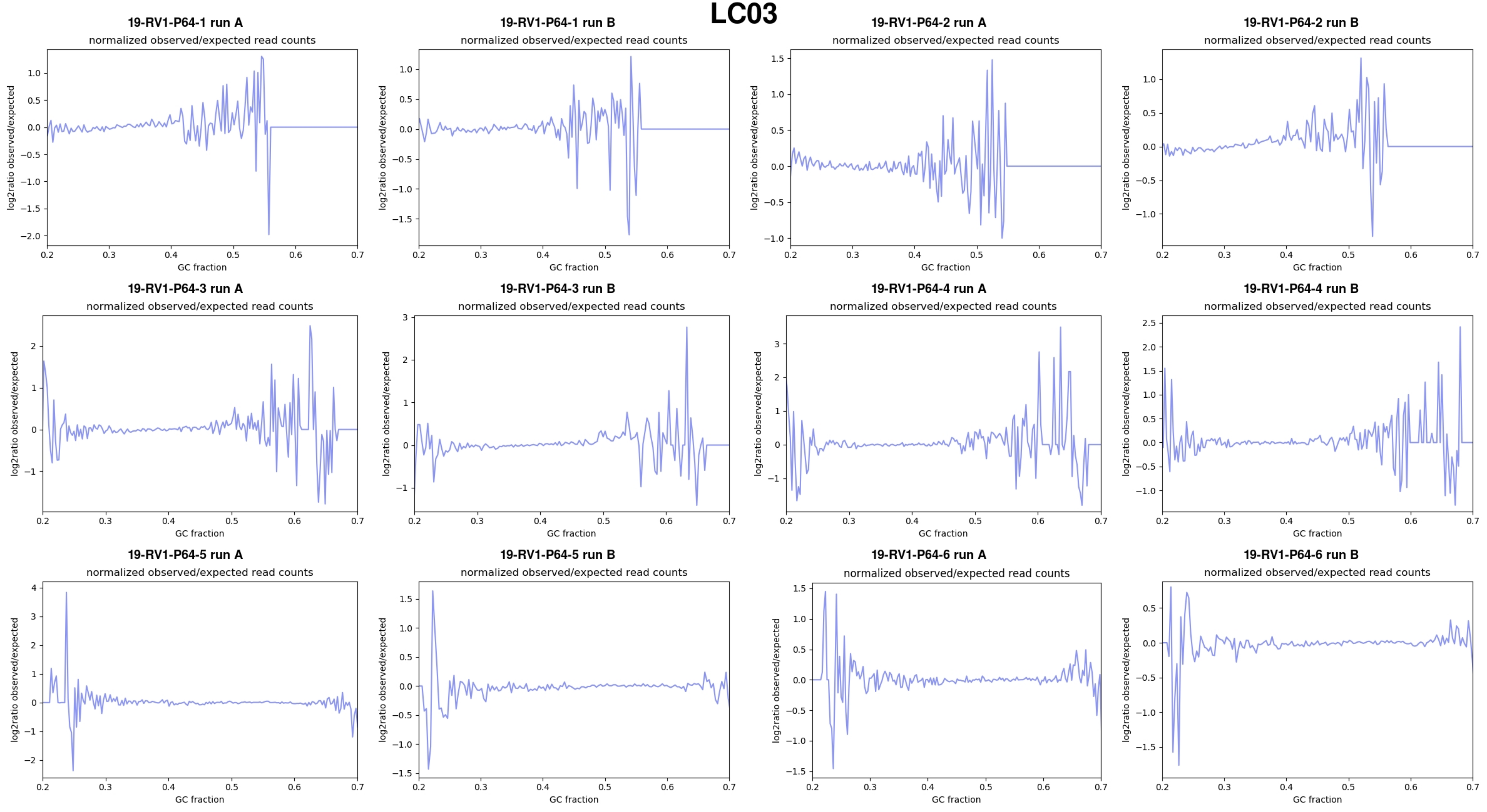
LC03
19-RV1-P64-1 run A
normalized observed/expected read counts
19-RV1-P64-1 run B
normalized observed/expected read counts
19-RV1-P64-2 run A
normalized observed/expected read counts
19-RV1-P64-2 run B
normalized observed/expected read counts
19-RV1-P64-3 run A
normalized observed/expected read counts
19-RV1-P64-3 run B
normalized observed/expected read counts
19-RV1-P64-4 run A
normalized observed/expected read counts
19-RV1-P64-4 run B
normalized observed/expected read counts
19-RV1-P64-5 run A
normalized observed/expected read counts
19-RV1-P64-5 run B
normalized observed/expected read counts
19-RV1-P64-6 run A
normalized observed/expected read counts
19-RV1-P64-6 run B
normalized observed/expected read counts
log2ratio observed/expected
GC fraction

LC04

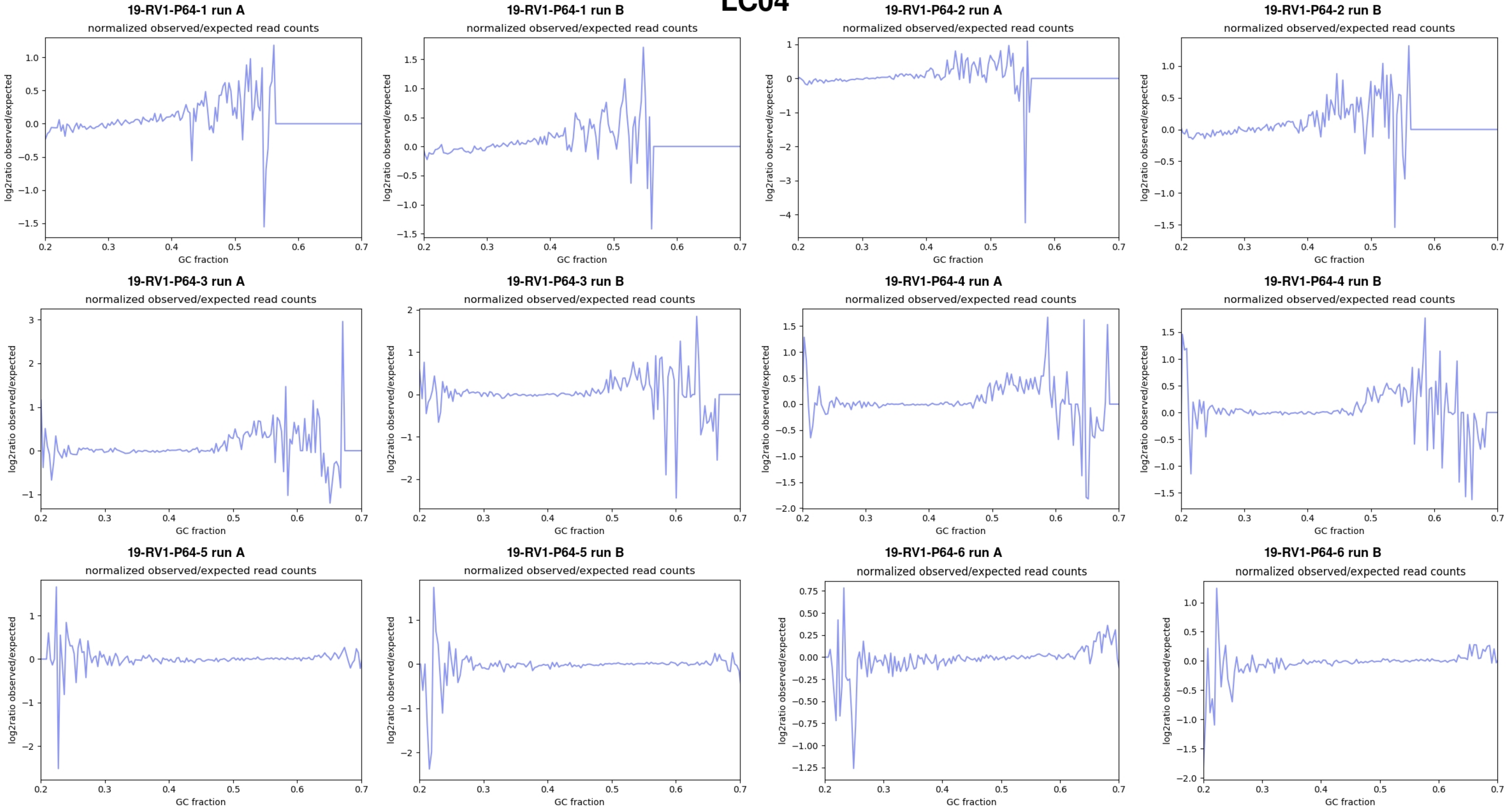
19-RV1-P64-1 run A
normalized observed/expected read counts
19-RV1-P64-1 run B
normalized observed/expected read counts
19-RV1-P64-2 run A
normalized observed/expected read counts
19-RV1-P64-2 run B
normalized observed/expected read counts
19-RV1-P64-3 run A
normalized observed/expected read counts
19-RV1-P64-3 run B
normalized observed/expected read counts
19-RV1-P64-4 run A
normalized observed/expected read counts
19-RV1-P64-4 run B
normalized observed/expected read counts
19-RV1-P64-5 run A
normalized observed/expected read counts
19-RV1-P64-5 run B
normalized observed/expected read counts
19-RV1-P64-6 run A
normalized observed/expected read counts
19-RV1-P64-6 run B
normalized observed/expected read counts
log2ratio observed/expected
GC fraction

LC05

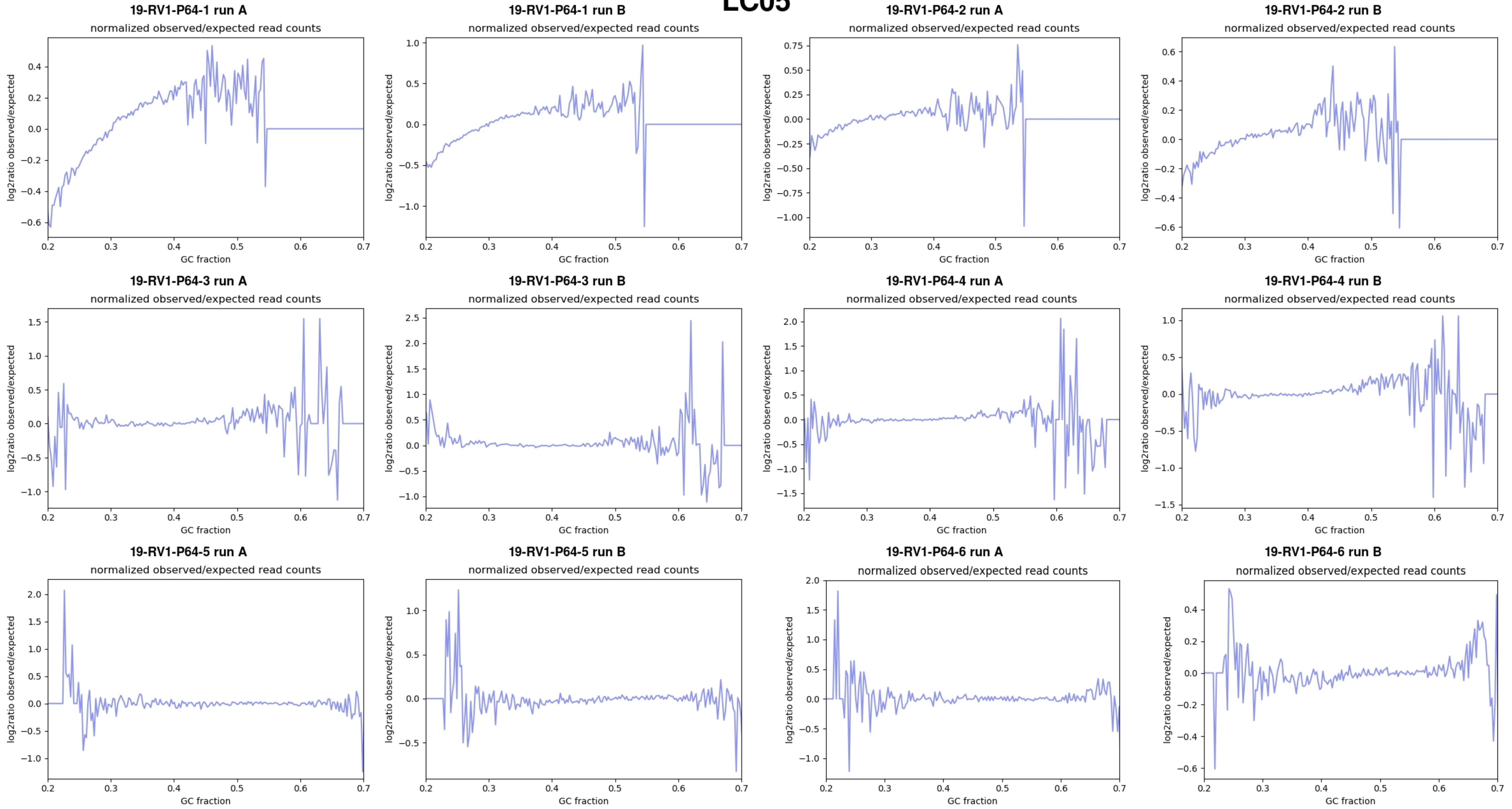
19-RV1-P64-1 run A
19-RV1-P64-1 run B
19-RV1-P64-2 run A
19-RV1-P64-2 run B
19-RV1-P64-3 run A
19-RV1-P64-3 run B
19-RV1-P64-4 run A
19-RV1-P64-4 run B
19-RV1-P64-5 run A
19-RV1-P64-5 run B
19-RV1-P64-6 run A
19-RV1-P64-6 run B
normalized observed/expected read counts
log2ratio observed/expected
GC fraction

LC06

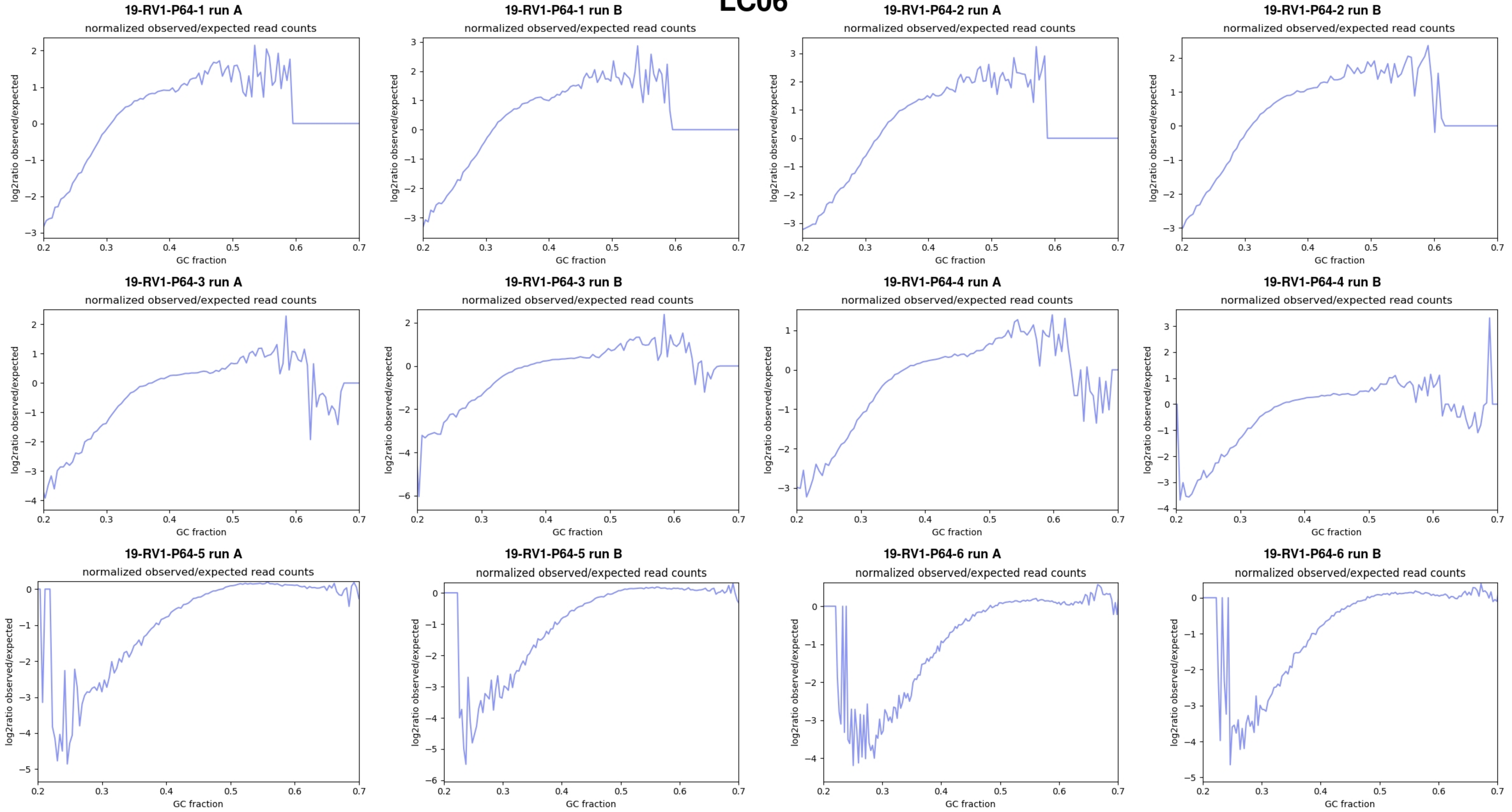

19-RV1-P64-1 run A
normalized observed/expected read counts
19-RV1-P64-1 run B
normalized observed/expected read counts
19-RV1-P64-2 run A
normalized observed/expected read counts
19-RV1-P64-2 run B
normalized observed/expected read counts
19-RV1-P64-3 run A
normalized observed/expected read counts
19-RV1-P64-3 run B
normalized observed/expected read counts
19-RV1-P64-4 run A
normalized observed/expected read counts
19-RV1-P64-4 run B
normalized observed/expected read counts
19-RV1-P64-5 run A
normalized observed/expected read counts
19-RV1-P64-5 run B
normalized observed/expected read counts
19-RV1-P64-6 run A
normalized observed/expected read counts
19-RV1-P64-6 run B
normalized observed/expected read counts
log2ratio observed/expected
GC fraction

LC07

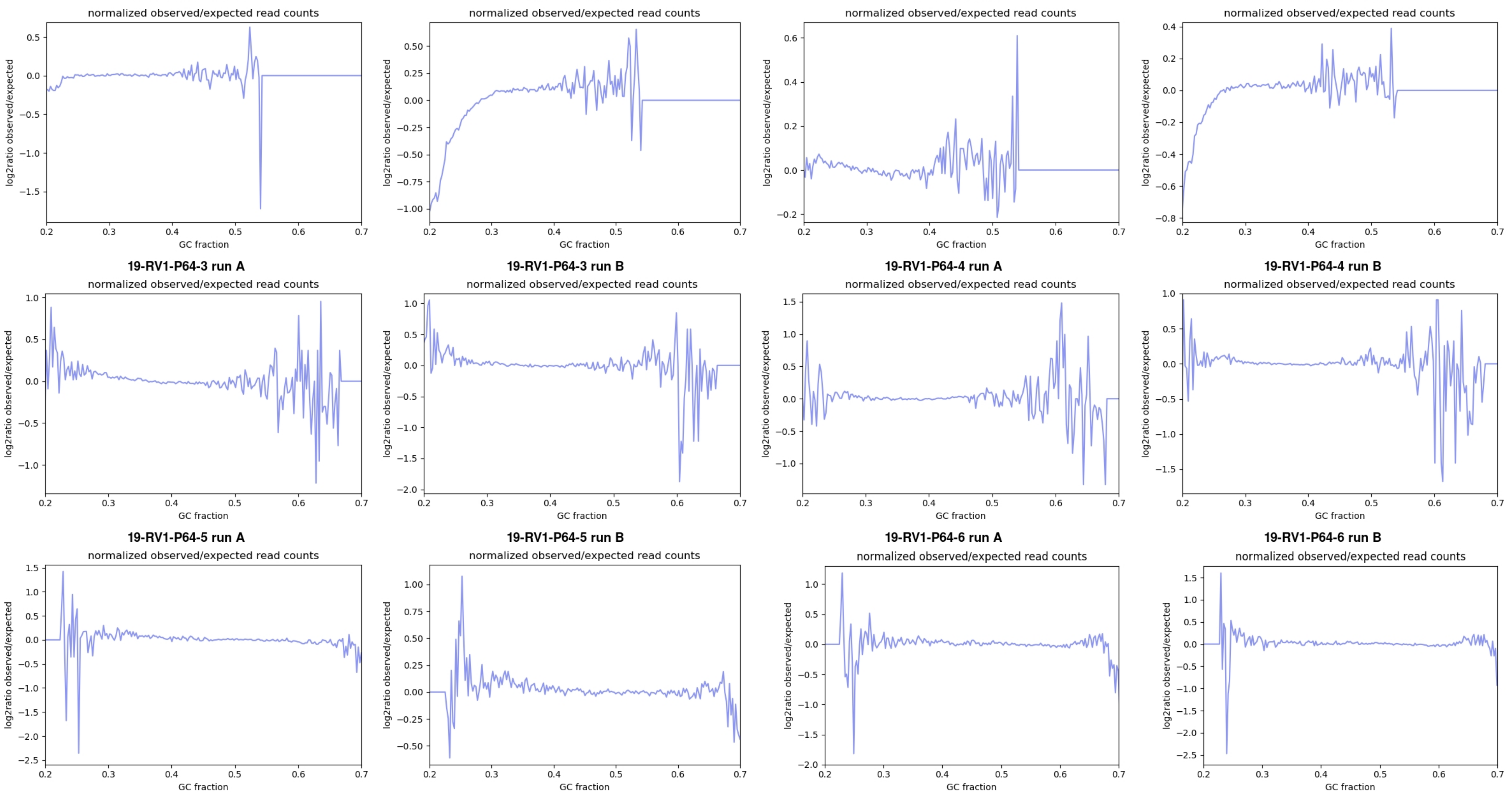
19-RV1-P64-1 run A
19-RV1-P64-1 run B
19-RV1-P64-2 run A
19-RV1-P64-2 run B
19-RV1-P64-3 run A
19-RV1-P64-3 run B
19-RV1-P64-4 run A
19-RV1-P64-4 run B
19-RV1-P64-5 run A
19-RV1-P64-5 run B
19-RV1-P64-6 run A
19-RV1-P64-6 run B
normalized observed/expected read counts
log2ratio observed/expected
GC fraction

LC08

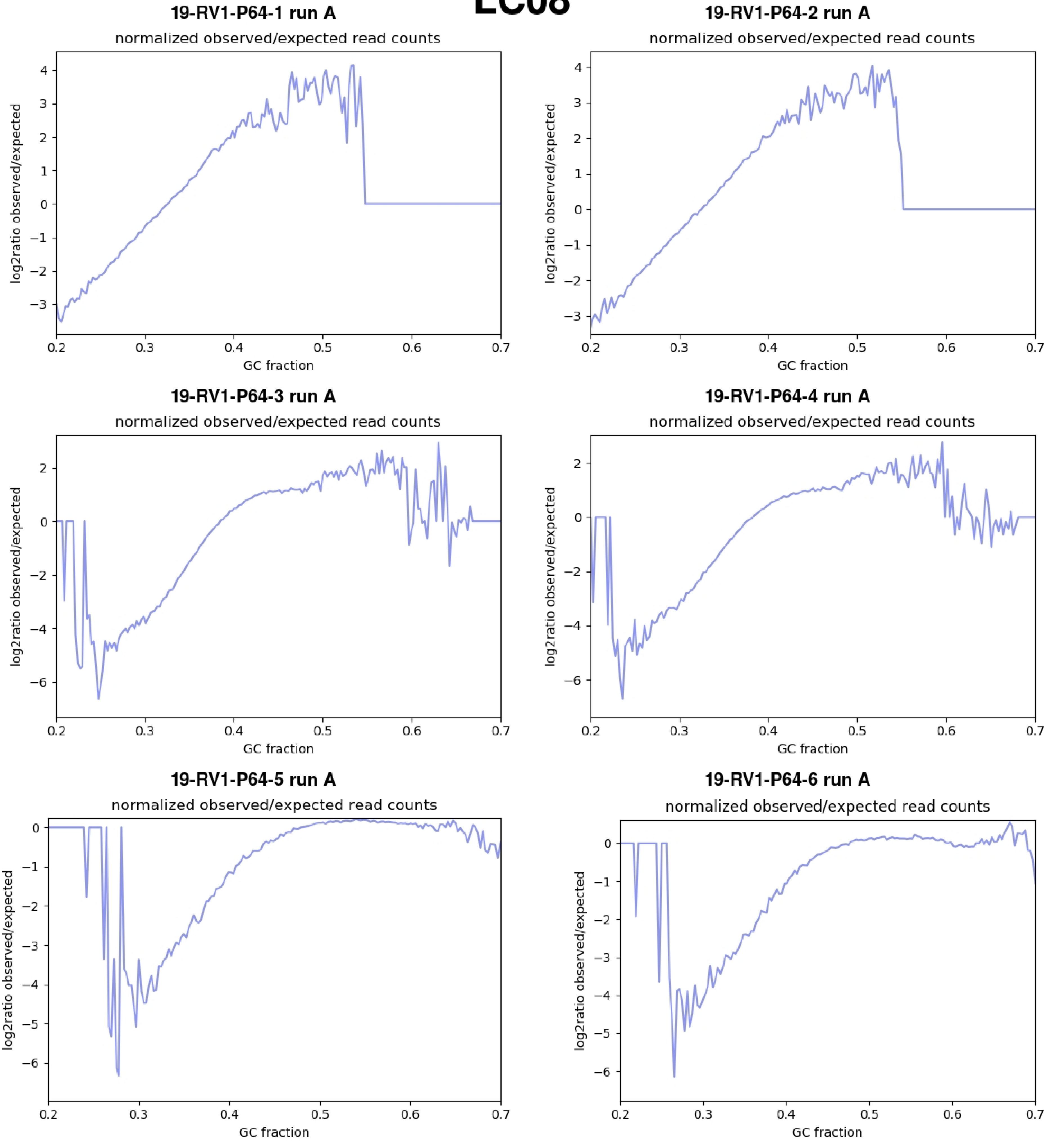
19-RV1-P64-1 run A
normalized observed/expected read counts
19-RV1-P64-2 run A
normalized observed/expected read counts
19-RV1-P64-3 run A
normalized observed/expected read counts
19-RV1-P64-4 run A
normalized observed/expected read counts
19-RV1-P64-5 run A
normalized observed/expected read counts
19-RV1-P64-6 run A
normalized observed/expected read counts
log2ratio observed/expected
GC fraction

LC09

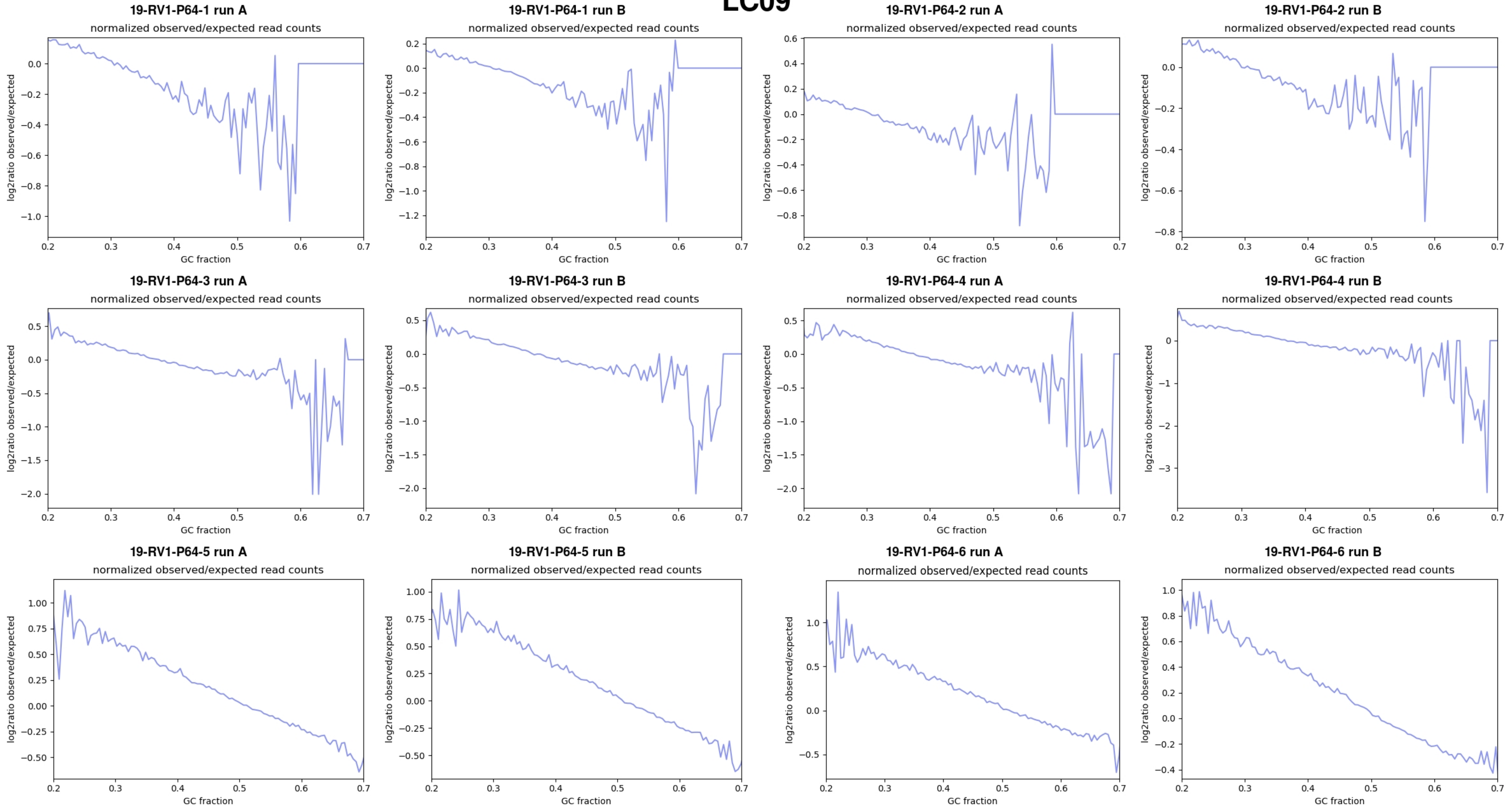

19-RV1-P64-1 run A
normalized observed/expected read counts
19-RV1-P64-1 run B
normalized observed/expected read counts
19-RV1-P64-2 run A
normalized observed/expected read counts
19-RV1-P64-2 run B
normalized observed/expected read counts
19-RV1-P64-3 run A
normalized observed/expected read counts
19-RV1-P64-3 run B
normalized observed/expected read counts
19-RV1-P64-4 run A
normalized observed/expected read counts
19-RV1-P64-4 run B
normalized observed/expected read counts
19-RV1-P64-5 run A
normalized observed/expected read counts
19-RV1-P64-5 run B
normalized observed/expected read counts
19-RV1-P64-6 run A
normalized observed/expected read counts
19-RV1-P64-6 run B
normalized observed/expected read counts
log2ratio observed/expected
GC fraction

LC10

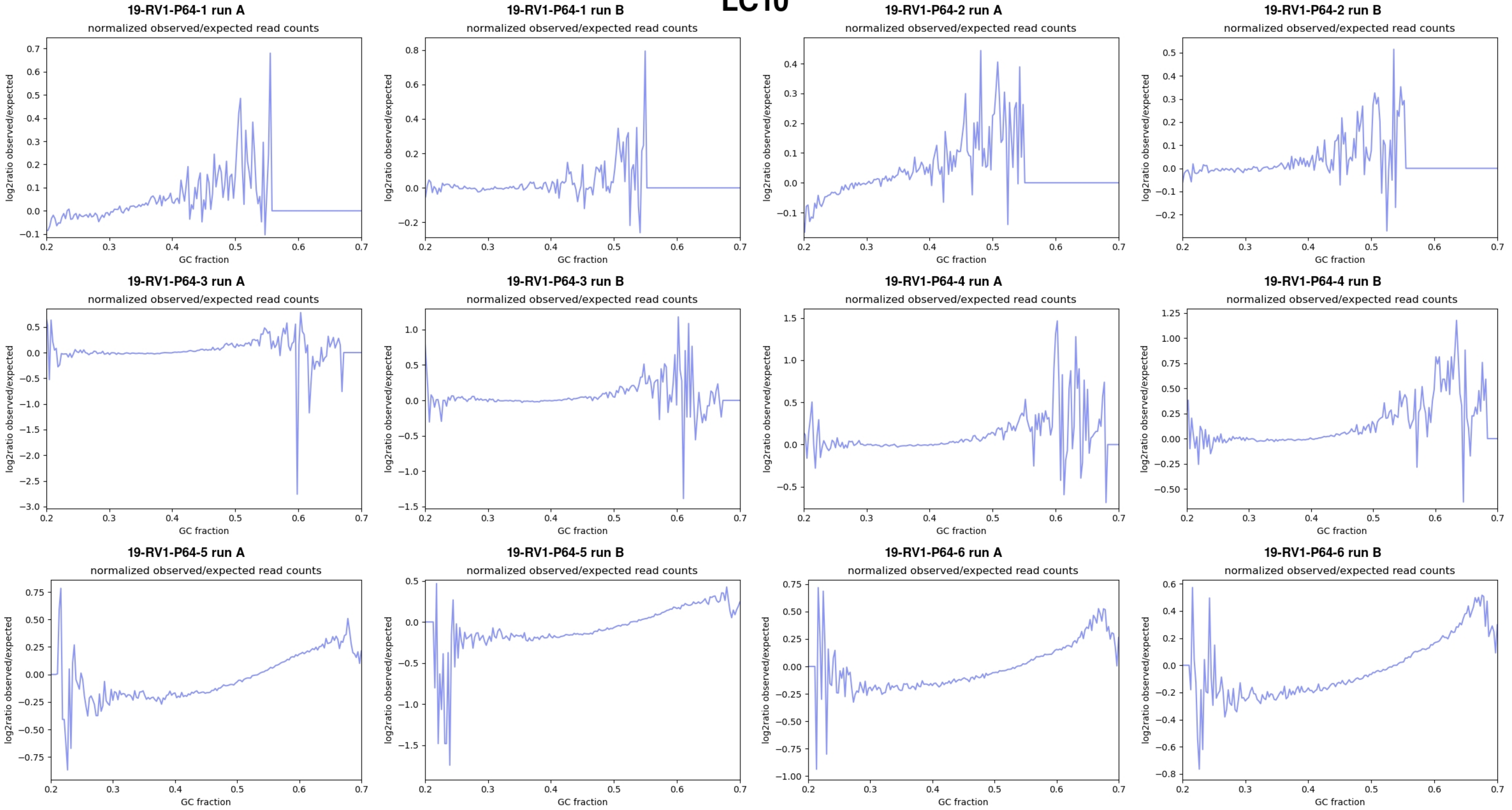
19-RV1-P64-1 run A
normalized observed/expected read counts
19-RV1-P64-1 run B
normalized observed/expected read counts
19-RV1-P64-2 run A
normalized observed/expected read counts
19-RV1-P64-2 run B
normalized observed/expected read counts
19-RV1-P64-3 run A
normalized observed/expected read counts
19-RV1-P64-3 run B
normalized observed/expected read counts
19-RV1-P64-4 run A
normalized observed/expected read counts
19-RV1-P64-4 run B
normalized observed/expected read counts
19-RV1-P64-5 run A
normalized observed/expected read counts
19-RV1-P64-5 run B
normalized observed/expected read counts
19-RV1-P64-6 run A
normalized observed/expected read counts
19-RV1-P64-6 run B
normalized observed/expected read counts
log2ratio observed/expected
GC fraction
