# Supplementary figures and images for: German-Wide Interlaboratory Study Compares Consistency, Accuracy and Reproducibility of Whole-Genome Short Read Sequencing
Source: Front Microbiol. 2020 Sep 11;11:573972. doi: 10.3389/fmicb.2020.573972 (PMC7516015; doi:10.3389/fmicb.2020.573972)

# effect of allele length threshold on cgMLST calling

**A**

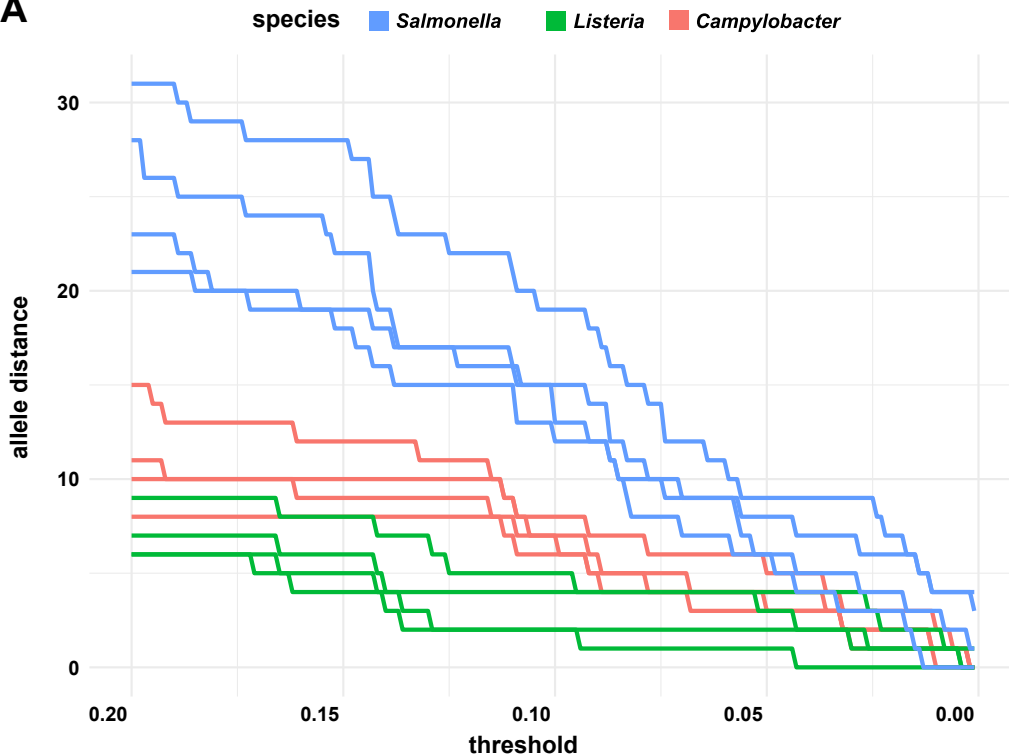

**B**

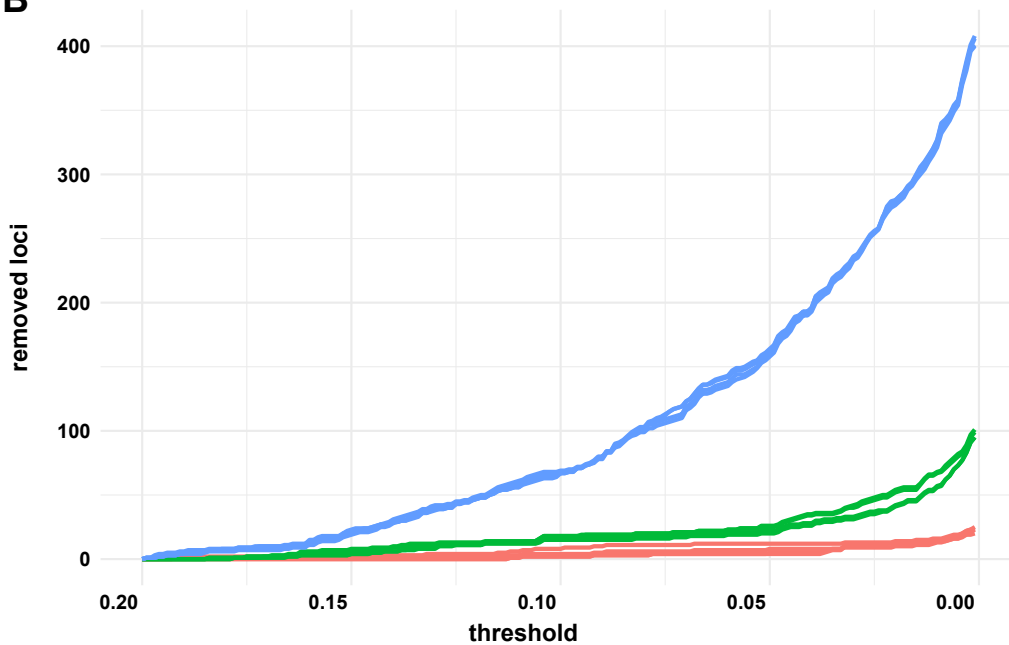

Supplement: FILE S1 — Information about the strains used for the interlaboratory study. [file Data_Sheet_1.zip › Supplementary File 9.PDF]
